# Effect of auriculotherapy and intervention types on weight control: A systematic review and meta-analysis protocol

**DOI:** 10.1097/MD.0000000000016959

**Published:** 2019-08-23

**Authors:** Junpeng Yao, Liping Chen, Leixiao Zhang, Siyuan Zhou, Qianhua Zheng, Xiumei Feng, Xi You, Lin Zhang, Ying Li

**Affiliations:** a Acupuncture and Tuina School, Chengdu University of Traditional Chinese Medicine; b The third affiliated hospital of Chengdu University of Traditional Chinese Medicine, Diabetes Mellitus Prevention and Control Center of Sichuan Province, Chengdu, Sichuan, China.

**Keywords:** auriculotherapy, meta-analysis, obesity, overweight, protocol, systematic review

## Abstract

**Background:**

Overweight and obesity characterized by abnormal or excessive fat accumulation, can cause many complications. Auriculotherapy, as the traditional Chinese technique, is widely applied in clinical trials for the management of body weight. The program aims to evaluate the effect and safety of auriculotherapy therapy and intervention types on weight control.

**Methods:**

All randomized controlled trials related to auriculotherapy targeting overweight and obesity will be searched in online databases, such as Medline, EMbase, Cochrane Central Register of Controlled Trials, AMED, CBM, Wanfang Data, and other databases from their inception to July 2019. The primary outcome is the difference in BMI from baseline to the end of studies. Secondary outcomes include the change of weight, percentage of body fat, waist circumference, serum lipid before and after treatment. Study selection, data extraction, and assessment of risk of bias will be performed independently by 2 reviewers. Comprehensive Meta-Analysis software (Version 3; Biostat Inc.) will be used for data synthesis.

**Results:**

This study will provide a comprehensive review of the available evidence for the treatment of obesity with auriculotherapy.

**Conclusion:**

The conclusion of this study will provide evidence to judge whether auriculotherapy is an effective therapeutic intervention for obesity.

**PROSPERO registration number:**

CRD42019136827.

## 1 Introduction

Overweight and obesity is a condition characterized by abnormal or excessive fat accumulation.^[1]^ According to the World Health Organization (WHO) standard for Asians, people were classified as obese (BMI ≥ 25 kg/m^2^), overweight (23 kg/m^2^ ≤ BMI < 25 kg/m^2^), and normal weight (18.5 kg/m^2^ ≤ BMI < 23 kg/m^2^).^[1,2]^ Worldwide, obesity is gradually becoming a major public health challenge. According to the epidemiological data, the prevalence of obese people has increased nearly three times since 1975.^[3,4]^ In 2014, the number of obese populations in China is the largest in the world, even for morbid obesity, the number comes to the second rank.^[5]^

Obesity can increase the incidence of many chronic conditions, such as diabetes, hypertension, cardiovascular diseases, and obstructive sleep apnea syndrome.^[6–8]^ Obesity is also associated with the mortality.^[9–11]^ It is estimated that about 4 million people died of overweight in 2015.^[3]^ There are high direct and indirect health care costs for treating obesity. According to a survey, the economic loss of the world's overweight-related diseases in 2014 was estimated at $200 million.^[12]^ At present, obesity has become the fourth world's major medical and social problems alongside AIDS, alcohol addiction and poison paralysis, and is the fifth largest risk of death worldwide.^[9,13]^ Therefore, weight control is an important public health issue that needs to be addressed to improve the health of individuals and medical expenditures.

Currently, treatment options for obesity mainly include calorie restriction, exercise, lifestyle modification as well as medications and bariatric surgery.^[14,15]^ The US Food and Drug Administration (FDA) has approved 5 weight loss drugs (orlistat, lorcaserin, naltrexone-bupropion, phentermine-topiramate, and liraglutide) for long-term management in obese or overweight individuals.^[16]^ However, the side effects and instability limit the clinical application of these drugs, such as phentermine can cause insomnia, orlistat causes diarrhea, phentermine-topiramate can bring paresthesia, dizziness, taste disturbance, use liraglutide can cause nausea and vomiting.^[17–20]^ Bariatric surgery may be the only long-term weight control therapy for the patient with morbid obesity but is hindered by heavily cost and possible postoperative complications such as anastomotic leakage and malnutrition.^[21,22]^ As a result, more and more overweight and obese patients are seeking cheaper, more convenient and less side effects of complementary and alternative therapies.

Auriculotherapy, also called auricular acupoint pressure or ear stimulation, is a method of diagnosing and treating diseases by stimulation of specific acupoints on the external ear, includes electrical stimulation, acupoint acupressure, different type of needles, seeds, and magnetic balls.^[23]^ Since it is thought that different auricular regions correspond to particular somatotopic areas of the body, auriculotherapy has been used as an effective treatment option for certain internal diseases including obesity.^[24]^ According to traditional Chinese medicine (TCM) theory, obesity is related to the dysfunction of the spleen, stomach, and kidney. The main meridians of these organs are closely related to the ear. By stimulating corresponding auricular points, meridian, and collaterals could be activated to tonify and promote Qi, so as to regulate the function of the organs and reduce the weight. Experimental study suggests that auricular stimulation may be involved in several mechanisms of BW regulation, such as anorexigenic and orexigenic peptides, glucose metabolism, insulin resistance, lipid metabolism, and inflammatory markers.^[25–27]^ The auricle nerve vessels are the most abundant, especially in the ear cavity and triangle nest, once stimulates the vagus nerve can affect the insulin value and suppress the appetite to achieve the purpose of weight loss.^[25]^ Therefore, auriculotherapy for weight control has the support of TCM and western medicine theory.

Nowadays, there have been more and more clinical studies suggest that auriculotherapy has beneficial effects in the treatment of obesity.^[28,29]^ There have been some systematic reviews published focusing on auricular acupoint stimulation in the treatment of obesity, but all of them, did not limit the control group to sham auriculotherapy, which could have led to the overestimation of the auriculotherapy effect due to the placebo effect.^[30,31]^ Furthermore, these studies did not report the differences in the effect of auricular acupoint stimulation by different auriculotherapy types.^[32,33]^ To the best of our knowledge, there is no systematic review has evaluated the pure effect of auriculotherapy for obesity. Hence, we conduct this systematic review to objectively evaluate the effect of pure auriculotherapy with that of sham auriculotherapy in the treatment for obesity and to compare the effect size of the treatments by auriculotherapy type.

## 2 Methods

### 2.1 Study registration

PROSPERO registration number is CRD42019136827. This protocol is reported in compliance with the Preferred Reporting Items for Systematic Reviews and Meta-Analyses Protocols (PRISMA-P) statement guidelines.^[34]^ The review will be conducted in accordance with the PRISMA guidelines and follows the recommendations of the Cochrane Handbook for Systematic Reviews of Interventions.^[35,36]^ If we refine the procedures described in this protocol, we will update the record in the PROSPERO and disclose them in future publications related to this study.

### 2.2 Inclusion criteria for study selection

#### 2.2.1 Types of study

In order to evaluate the pure effect of auriculotherapy in the treatment of obesity, we will just include the randomized clinical trials (RCT) that compared auriculotherapy (auricular acupuncture or auricular acupressure or auricular acupuncture with electrical stimulation) with sham auriculotherapy or placebo or no treatment (waiting list control). Completed and ongoing trials will be included. Owing to the language restriction of our researchers, we will limit the language of search literature to Chinese and English. If the study was designed as a cross-over trial, only the first phase results will be analyzed in order to eliminate carry-over effects.

Trials will be excluded as follows:

(1) studies that involved a combination of auriculotherapy with other treatments, such as moxibustion, massage, cupping, and drugs or Chinese herbs;
(2) comparing auriculotherapy with other forms of acupuncture or other treatment;
(3) quasi-randomized trials and case reports;
(4) only providing the information of effective rate and not providing the data of BMI from baseline to the end of studies.

#### 2.2.2 Types of participants

Trials including patients who meet the diagnostic criteria of overweight or obese and age were greater than 18 years will be included. All eligible study participants will be included in this review regardless of their race or gender. Trials including participants who are not appropriate to receive auriculotherapy, such as pregnant or lactating women, and those were secondary obesity or with additional severe diseases will be excluded.

#### 2.2.3 Types of intervention

The experimental group should be treated with auriculotherapy including auricular acupuncture or auricular acupressure or auricular acupuncture with electrical stimulation, and acupoints used according to TCM nomenclature. The types of seed used and the duration of treatment will be unlimited. Auriculotherapy combined with other conventional therapy should be excluded.

To evaluate the true effect of auriculotherapy on obesity, we will limit the interventions in the control groups to sham auriculotherapy or no treatment or placebo. Studies involving auriculotherapy for weight a control but without control arm will be excluded. In addition, studies involving sham auricular acupuncture for weight control that was not masked from participants in the control group will also be excluded.

#### 2.2.4 Types of outcome measures

The primary outcome of effect is the difference in BMI from baseline to the end of studies. BMI is an index defined by one's weight in kilogram divided by height in meter square (kg/m^2^). The secondary outcomes include the change of weight, percentage of body fat (BFP), WC, serum lipid (such as cholesterol and triglyceride) before and after treatment, adverse events will also be measured as secondary outcomes for safety assessment.

### 2.3 Data sources

Our systematic review will search all RCTs for auriculotherapy on weight control, electronically and manually, regardless of publication status, till 31th July 2019. Online databases include Medline (via PubMed), EMbase, Cochrane Central Register of Controlled Trials, AMED, CBM, Wanfang Data, VIP, and CNKI. Ongoing trials with unpublished data will be retrieved from the four following clinical trial registries: International Clinical Trials Registry Platform (ICTRP), NIH clinical registry Clinical Trials.gov, the Chinese clinical registry, and the Australian New Zealand Clinical Trials Registry. The reference lists of the selected studies and published systematic reviews will be screened for additional studies. Manually search for the grey literature, including conference proceedings.

### 2.4 Search strategy

The search strategy will be followed the PRISMA guidelines. The key search terms are (“obesity” OR “overweight” OR “weight reduction”) AND (“auricular acupuncture” OR “auricular acupressure” OR “auricular pressing” OR “auricular needle” OR “auricular plaster”) AND (“randomized”). The search strategy will be adapted to different databases demands. Search strategy in PubMed is shown in Table 1.

**Table 1:**
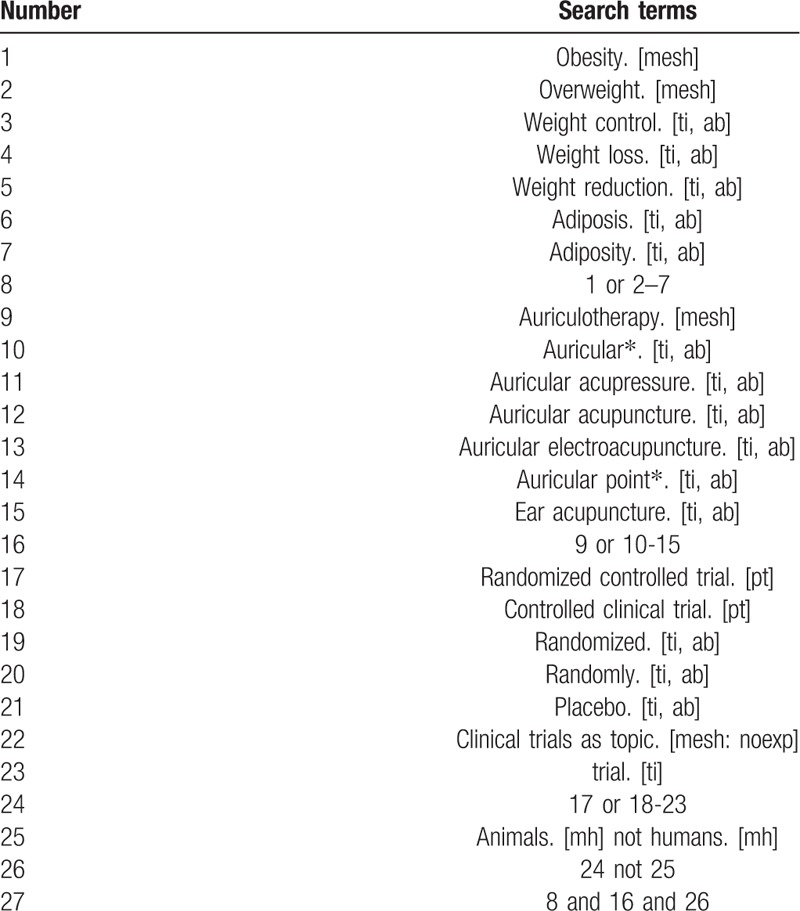
Search strategy used in PubMed database.

### 2.5 Data collection and analysis

#### 2.5.1 Selection of studies

**Figure 1:**
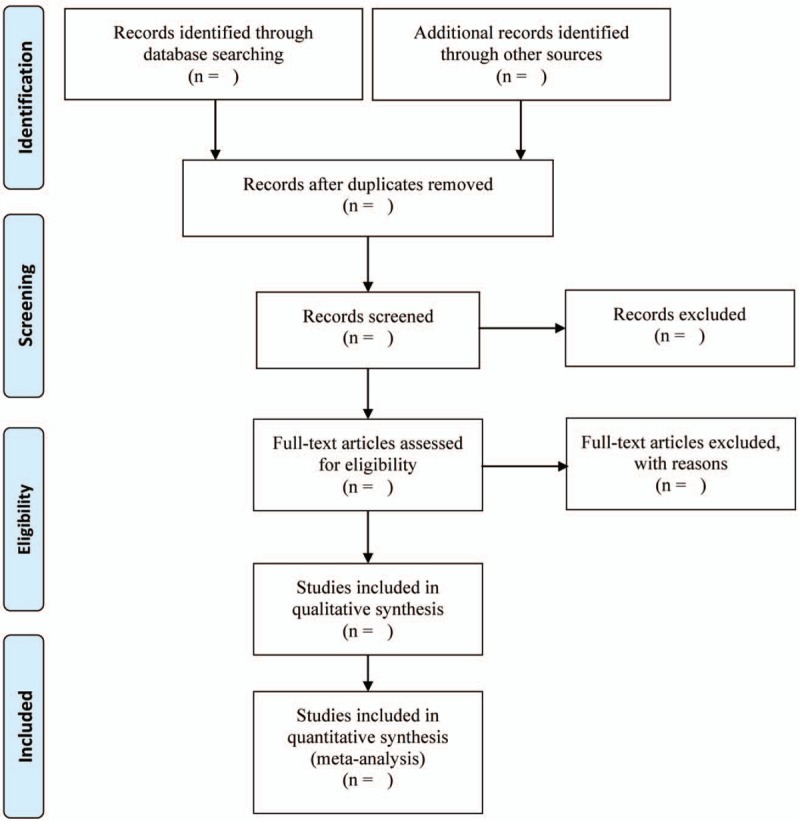
The PRISMA flow diagram of studies identified.

In the literature screening process, search results will be imported from the original databases to Endnote V.X9 and repetitive studies will be deleted by the software. Two reviewers will independently screen all retrieval research, read the title, abstract and keywords to determine which studies meet the inclusion criteria. We will obtain the full text of all relevant studies for further evaluation. Studies excluded after reading the full text will be recorded and explained. The selection results will be cross-checked by the 2 reviewers. Any disagreement will be resolved by consensus. Further argument will be arbitrated by a third reviewer. The primary selection process is shown in a PRISMA flow chart (Fig. 1).

#### 2.5.2 Data extraction and management

The following data will be extracted from the selected studies by 2 reviewers independently using a predefined data acquisition form: the characteristics of the study (publication year, nationality, journal, study design), participants (sample size, age, sex, height, weight, BMI), intervention (duration, frequency, types of auriculotherapy, types of control group), weight-related outcomes (the difference in BMI, the change of weight, BFP, waist circumference, and serum lipid), adverse reactions and other information. Any discrepancy noticed in the process of data extraction will be resolved through discussion and the suggestion of a third reviewer. For publications with insufficient or ambiguous data, we will attempt to obtain information from the corresponding authors by e-mail or telephone.

#### 2.5.3 Assessment of risk of bias and reporting of study quality

The authors will assess the study quality by using the checklist developed by the Cochrane Collaboration's bias risk assessment tool which evaluates the presence of potential selection bias (random sequence generation and allocation concealment), performance bias (blinding of investigators and participants), detection bias (blinding of outcome assessors), attrition bias (incomplete outcome data), reporting bias (selective reporting) and possible other sources of bias (funding bias).^[36]^ Risk bias of trials was graded as high, low or unclear risk. If inconsistent results appear, the final decisions will be made by the third author.

#### 2.5.4 Measures of treatment effect

Data analysis and quantitative data synthesis will be performed using Comprehensive Meta-Analysis software (Version 3; Biostat Inc.). For continuous data, we will use the standardized mean difference (SMD) along with its 95% confidence intervals to measure the therapeutic effect, whereas dichotomous data will be presented as relative risk (RR) with 95%CI for analysis.

#### 2.5.5 Assessment of heterogeneity

Heterogeneity between studies will be assessed using the *I*^2^ test. The study is not considered to have large heterogeneous if the *I*^2^ test is less than 50%. However, when the *I*^2^ values higher than 50%, there is substantial heterogeneity among the trials, we will search for possible causes from a clinical and methodological perspective, and provide a descriptive analysis or subgroup analysis to explore the possible causes of heterogeneity. Meanwhile, the corresponding *P* values will also be taken into account. The results will be discussed according to the different heterogeneity.

#### 2.5.6 Assessment of reporting biases

We will use funnel charts to visually inspect publication biases. If a sufficient number of included studies (more than 10 trials) are available, the funnel plot will be used to assess the reported bias. If the funnel plot is found to be asymmetrical, analyze the cause using Egger regression test. We will include all eligible trials regardless of the quality of the method.

#### 2.5.7 Data synthesis

We will use Comprehensive Meta-Analysis software (Version 3; Biostat Inc.) for all statistical analysis. In order to maximize information, data on outcomes reported by single studies or in a descriptive way will be reported narratively. If the *I*^2^ test is less than 50%, the fixed effects model will be used for data synthesis. If considerable heterogeneity is observed, data will be pooled using the random-effects model.

#### 2.5.8 Subgroup analysis

In the present study, the heterogeneity will significant with respect to the auriculotherapy types, subjects, treatment period, etc. Therefore, subgroup analysis will be employed according to different types of auriculotherapy, the initial BMI of patients, different treatment duration, or frequency, different control groups (sham auriculotherapy or placebo), different outcomes, and so on.

#### 2.5.9 Sensitivity analysis

Multiple sensitivity analysis will be performed to assess the robustness of the summary estimates and to detect if any particular study accounted for a large proportion of heterogeneity. These will be based on different statistical approach, different heterogeneity quality and different sample size. The meta-analysis will be reused, and more inferior quality studies will be excluded. The results will be compared and discussed according to the results.

#### 2.5.10 Grading the quality of evidence

The GRADE approach will be used to rate the quality of evidence of estimates derived from this study.^[37]^ In this approach, direct evidence from RCTs starts at high quality and can be downgraded based on the risk of bias, indirectness, imprecision, inconsistency (or heterogeneity), and/or publication bias to levels of moderate, low, and very low quality.^[38]^

## 3 Discussion

Overweight and obese problem is a pandemic public health issue in the world, with more medical costs and seriously affect the quality of people's life. Therefore, several interventions have been explored, such as lifestyle intervention, pharmaceutical, and bariatric surgery treatments. People can reduce 5% to 10% of initial weight through intensive lifestyle intervention by changes diet and physical activity,^[39,40]^ but long-term weight maintenance is difficult. Meanwhile, pharmaceutical and bariatric surgery treatments are effective for some overweight and obese people but are expensive and often accompanied by adverse side effects.

With the development of complementary and alternative medicine, auriculotherapy has been widely applied in clinic especially in Asian countries. And it is widely used in the regulation of obesity. As mentioned in the preceding texts, the previous reviews did not separate the pure effect of auriculotherapy from other interventions and sham auriculotherapy. Therefore, the effect of auriculotherapy may have been overestimated in the meta-analysis due to the placebo effect. In this study, we will objectively evaluate the pure effect of auriculotherapy for obesity, and also examined the effect of auriculotherapy on obesity by different types, hoping to provide convincing evidence for patients and clinicians during the decision-making process.

## Author contributions

**Conceptualization:** Junpeng Yao, Lin Zhang.

**Data curation:** Liping Chen, Siyuan Zhou, Qianhua Zheng.

**Formal analysis:** Leixiao Zhang, Qianhua Zheng.

**Funding acquisition:** Lin Zhang.

**Investigation:** Xi You.

**Methodology:** Junpeng Yao, Xiumei Feng, Xi You.

**Software:** Siyuan Zhou, Lin Zhang.

**Supervision:** Ying Li.

**Writing – original draft:** Junpeng Yao, Liping Chen.

**Writing – review & editing:** Ying Li, Leixiao Zhang.

## References

[1] World Health Organization. Obesity and Overweight Fact Sheet. Available at: https://www.who.int/en/news-room/fact-sheets/detail/obesity-and-overweight [access date March 1, 2019].

[2] Gadde KM Martin CK Berthoud HR Obesity: pathophysiology and management. J Am Coll Cardiol 2018;71:69–84. 29301630 10.1016/j.jacc.2017.11.011 PMC7958889

[3] Afshin A Forouzanfar MH Reitsma MB Health effects of overweight and obesity in 195 countries over 25 years. N Engl J Med 2017;377:13–27. 28604169 10.1056/NEJMoa1614362 PMC5477817

[4] Collaboration NCDRF. Trends in adult body-mass index in 200 countries from 1975 to 2014: a pooled analysis of 1698 population-based measurement studies with 19.2 million participants. Lancet 2016; 387:1377–96. 27115820 10.1016/S0140-6736(16)30054-X PMC7615134

[5] Yang ZJ Liu J Ge JP The Obesity Expert Panel. Prevalence of cardiovascular disease risk factor in the Chinese population: the 2007–2008 China national diabetes and metabolic disorders study. Eur Heart J 2012;33:213–20. 21719451 10.1093/eurheartj/ehr205

[6] Jehan S Myers AK Zizi F Obesity, obstructive sleep apnea and type 2 diabetes mellitus: epidemiology and pathophysiologic insights. Sleep Med Disord 2018;2:52–8. 30167574 PMC6112821

[7] Kyrgiou M Kalliala I Markozannes G Adiposity and cancer at major anatomical sites: umbrella review of the literature. BMJ 2017;356:j477. 28246088 10.1136/bmj.j477 PMC5421437

[8] Field AE Coakley EH Must A Impact of overweight on the risk of developing common chronic diseases during a 10-year period. Arch Intern Med 2001;161:1581–6. 11434789 10.1001/archinte.161.13.1581

[9] Grover SA Kaouache M Rempel P Years of life lost and healthy life-years lost from diabetes and cardiovascular disease in overweight and obese people: a modelling study. Lancet Diabetes Endocrinol 2015;3:114–22. 25483220 10.1016/S2213-8587(14)70229-3

[10] Poobalan AS Aucott LS Smith WC Long-term weight loss effects on all cause mortality in overweight/obese populations. Obes Rev 2007;8:503–13. 17949355 10.1111/j.1467-789X.2007.00393.x

[11] Flegal KM Kit BK Orpana H Association of all-cause mortality with overweight and obesity using standard body mass index categories: a systematic review and meta-analysis. JAMA 2013;309:71–82. 23280227 10.1001/jama.2012.113905 PMC4855514

[12] Lauby-Secretan B Scoccianti C Loomis D Body fatness and cancer--viewpoint of the IARC working group. N Engl J Med 2016;375:794–8. 27557308 10.1056/NEJMsr1606602 PMC6754861

[13] Kolotkin RL Andersen JR A systematic review of reviews: exploring the relationship between obesity, weight loss and health-related quality of life. Clin Obes 2017;7:273–89. 28695722 10.1111/cob.12203 PMC5600094

[14] Jensen MD Ryan DH Apovian CM 2013 AHA/ACC/TOS guideline for the management of overweight and obesity in adults: a report of the American College of Cardiology/American Heart Association Task Force on Practice Guidelines and The Obesity Society. J Am Coll Cardiol 2014;63:2985–3023. 24239920 10.1016/j.jacc.2013.11.004

[15] The Obesity Expert Panel. Executive summary: Guidelines (2013) for the management of overweight and obesity in adults: a report of the American College of Cardiology/American Heart Association Task Force on Practice Guidelines and the Obesity Society published by the Obesity Society and American College of Cardiology/American Heart Association Task Force on Practice Guidelines. Based on a systematic review from the The Obesity Expert Panel, 2013. Obesity (Silver Spring) 2014; 22 Suppl 2:S5–S39. 10.1002/oby.20821 24961825

[16] Srivastava G Apovian C Future pharmacotherapy for obesity: new anti-obesity drugs on the horizon. Curr Obes Rep 2018;7:147–61. 29504049 10.1007/s13679-018-0300-4

[17] Padwal RS Majumdar SR Drug treatments for obesity: orlistat, sibutramine, and rimonabant. Lancet 2007;369:71–7. 17208644 10.1016/S0140-6736(07)60033-6

[18] Witkamp RF Current and future drug targets in weight management. Pharm Res 2011;28:1792–818. 21181547 10.1007/s11095-010-0341-1 PMC3130908

[19] Kakkar AK Dahiya N Drug treatment of obesity: current status and future prospects. Eur J Intern Med 2015;26:89–94. 25634851 10.1016/j.ejim.2015.01.005

[20] Khera R Murad MH Chandar AK Association of pharmacological treatments for obesity with weight loss and adverse events: a systematic review and meta-analysis. JAMA 2016;315:2424–34. 27299618 10.1001/jama.2016.7602 PMC5617638

[21] Kassir R Debs T Blanc P Complications of bariatric surgery: presentation and emergency management. Int J Surg 2016;27:77–81. 26808323 10.1016/j.ijsu.2016.01.067

[22] Goto T Hirayama A Faridi MK Association of bariatric surgery with risk of infectious diseases: a self-controlled case series analysis. Clin Infect Dis 2017;65:1349–55. 28637274 10.1093/cid/cix541

[23] Wu C Liu P Fu H Transcutaneous auricular vagus nerve stimulation in treating major depressive disorder: A systematic review and meta-analysis. Medicine (Baltimore) 2018;97:e13845. 30593183 10.1097/MD.0000000000013845 PMC6314717

[24] Belivani M Dimitroula C Katsiki N Acupuncture in the treatment of obesity: a narrative review of the literature. Acupunct Med 2013;31:88–97. 23153472 10.1136/acupmed-2012-010247

[25] Li H Zhang JB Xu C Effects and mechanisms of auricular vagus nerve stimulation on high-fat-diet--induced obese rats. Nutrition 2015;31:1416–22. 26429664 10.1016/j.nut.2015.05.007

[26] Chen SH Chen HC Hsieh CL Electric stimulation of ears accelerates body weight loss mediated by high-fat to low-fat diet switch accompanied by increased white adipose tissue browning in C57BL/6 J mice. BMC Complement Altern Med 2018;18:323. 30518367 10.1186/s12906-018-2388-1 PMC6282328

[27] Shiraishi T Onoe M Kojima T Effects of auricular stimulation on feeding-related hypothalamic neuronal activity in normal and obese rats. Brain Res Bull 1995;36:141–8. 7895091 10.1016/0361-9230(94)00179-5

[28] Kim SY Shin IS Park YJ Effect of acupuncture and intervention types on weight loss: a systematic review and meta-analysis. Obes Rev 2018;19:1585–96. 30180304 10.1111/obr.12747

[29] Hsieh CH Su TJ Fang YW Effects of auricular acupressure on weight reduction and abdominal obesity in Asian young adults: a randomized controlled trial. Am J Chin Med 2011;39:433–40. 21598412 10.1142/S0192415X11008932

[30] Cho SH Lee JS Thabane L Acupuncture for obesity: a systematic review and meta-analysis. Int J Obes (Lond) 2009;33:183–96. 19139756 10.1038/ijo.2008.269

[31] Zhang RQ Tan J Li FY Acupuncture for the treatment of obesity in adults: a systematic review and meta-analysis. Postgrad Med J 2017;93:743–51. 28689171 10.1136/postgradmedj-2017-134969

[32] Huang CF Guo SE Chou FH Auricular acupressure for overweight and obese individuals: a systematic review and meta-analysis. Medicine (Baltimore) 2019;98:e16144. 31261540 10.1097/MD.0000000000016144 PMC6617497

[33] Yeh TL Chen HH Pai TP The effect of auricular acupoint stimulation in overweight and obese adults: a systematic review and meta-analysis of randomized controlled trials. Evid Based Complement Alternat Med 2017;2017:3080547. 29358964 10.1155/2017/3080547 PMC5735786

[34] Shamseer L Moher D Clarke M Preferred reporting items for systematic review and meta-analysis protocols (PRISMA-P) 2015: elaboration and explanation. BMJ 2015;350:g7647. 25555855 10.1136/bmj.g7647

[35] Liberati A Altman DG Tetzlaff J The PRISMA statement for reporting systematic reviews and meta-analyses of studies that evaluate health care interventions: explanation and elaboration. J Clin Epidemiol 2009;62:e1–34. 19631507 10.1016/j.jclinepi.2009.06.006

[36] Deeks JJ Higgins JPT Altman DG Green S Cochrane handbook for systematic reviews of interventions version 5.1. 0 (updated March 2011). The Cochrane Collaboration, 2011.

[37] Schunemann HJ Oxman AD Brozek J Grading quality of evidence and strength of recommendations for diagnostic tests and strategies. BMJ 2008;336:1106–10. 18483053 10.1136/bmj.39500.677199.AE PMC2386626

[38] van de Griendt EJ Tuut MK de Groot H Applicability of evidence from previous systematic reviews on immunotherapy in current practice of childhood asthma treatment: a GRADE (Grading of Recommendations Assessment, Development and Evaluation) systematic review. BMJ Open 2017;7:e16326. 10.1136/bmjopen-2017-016326 PMC5770836 29288175

[39] Manna P Jain SK Obesity, oxidative stress, adipose tissue dysfunction, and the associated health risks: causes and therapeutic strategies. Metab Syndr Relat Disord 2015;13:423–44. 26569333 10.1089/met.2015.0095 PMC4808277

[40] Stephens SK Cobiac LJ Veerman JL Improving diet and physical activity to reduce population prevalence of overweight and obesity: an overview of current evidence. Prev Med 2014;62:167–78. 24534460 10.1016/j.ypmed.2014.02.008

